# The Role of Alarmins in the Pathogenesis of Asthma

**DOI:** 10.3390/biom15070996

**Published:** 2025-07-11

**Authors:** Paulina Plewa, Julia Pokwicka, Estera Bakinowska, Kajetan Kiełbowski, Andrzej Pawlik

**Affiliations:** Department of Physiology, Pomeranian Medical University, 70-111 Szczecin, Poland; paulina.plewa@op.pl (P.P.); jpokwicka@gmail.com (J.P.); esterabakinowska@gmail.com (E.B.)

**Keywords:** asthma, alarmins, HMGB1, S100 proteins, interleukin-33

## Abstract

Asthma is defined as a chronic respiratory disease, the processes of which are mainly related to the hyperreactivity of the immune system. Airway hyperresponsiveness and remodeling are other hallmarks of asthma that are strongly involved in the progression of the disease. Moreover, asthma is associated with the occurrence of atopic dermatitis, chronic sinusitis, allergic rhinitis, and a high profile of T2-type cytokines, such as IL-4, IL-5 and IL-13. The hyperresponsiveness of the immune system is a consequence of aberrant levels of alarmins, endogenous molecules that induce pro-inflammatory responses. They are released as a result of a defect or cell death, leading to the initiation of an inflammatory reaction. High-mobility group box 1 (HMGB1), S100 proteins, interleukin-33 (IL-33), thymic stromal lymphopoietin (TSLP), and IL-25 bind to various receptors, influencing the behavior of immune cells, resulting in stimulated migration and activation of these cells. In this review, we will discuss the potential role of alarmins in the pathogenesis of asthma.

## 1. Introduction

Asthma is a chronic airway disease typically manifested as wheezing, dyspnea, cough, and a tendency for exacerbations. It is estimated that approximately 300 million individuals suffer from asthma globally [1]. The disease is associated with the presence of complications and links to other diseases, such as gastroesophageal reflux, cardiovascular disease, and obesity, among others [2]. Asthma is a very heterogeneous condition, with multiple underlying processes leading to this disease. Therefore, several asthma phenotypes can be differentiated [3]. The two main endotypes of asthma involve T2-high and T2-low (or non-T2). T2-high category covers conditions caused by inflammatory pathways associated with elevated levels of T2 cytokines, involving TSLP, IL-33 and IL-25, among others. Those pathways mainly lead to activation of eosinophils; thus, T2-high asthma can also be referred to as eosinophilic asthma. Atopic asthma represents an example of the T2-high subtype; symptoms develop after an individual is exposed to allergens. The T2-low category consists of instances when T2 cytokine levels are not elevated. Patients with non-T2 asthma present mainly neutrophilic or paucigranulocytic inflammation; thus, neutrophilic asthma is a T2-low type of this disease. Biomarkers associated with this group are TNF-α, IL-1β, IL-6, IL-8, IL-17, Th1, and Th17. Patients with this type of asthma do not develop symptoms after contact with allergens; their symptoms are caused by other factors. For example, obesity related asthma belongs in this category, as it is associated with neutrophilic inflammation [3,4,5,6]. Management of asthma involves several potential therapeutics, including glucocorticosteroids, short-acting beta agonists, antileukotriene agents and biologics [7]. The latter group is composed of monoclonal antibodies, such as benralizumab (anti-IL-5), dupilumab (anti-IL-4), mepolizumab (anti-IL-5), omalizumab (anti-IgE), and reslizumab (anti-IL-5), which are approved for the treatment of severe asthma [8].

Since there is a strong role of the immune system in asthma, molecules that induce inflammatory responses, known as alarmins, are thought to be involved in the pathogenesis of asthma. Alarmins represent a broad group of molecules that can be classified according to their source of origin. Defensins and cathelicidin belong to the granule-derived subgroup, high-mobility group box-1 (HMGB1) and high-mobility group nucleosome-binding domain 1 (HMGN1) proteins are located in the nucleus, while the S100 and heat shock proteins (HSPs) are cytoplasmic alarmins [9]. As the name suggests, the aim of alarmins is to alert the immune system and induce an indirect or direct adaptive immune system. They are endogenous molecules released from cells through the secretion pathways or during non-programmed cell death [10]. Depending on the molecule, alarmins can interact with various receptors, such as the receptor for advanced glycation endproducts (RAGE) and toll-like receptors (TLRs). Stimulation of TLRs promotes the activity of the major pro-inflammatory mediator nuclear factor kappa B (NF-κB).

The involvement of alarmins in the pathogenesis of inflammatory conditions has been discussed previously. Studies demonstrate abnormal levels of alarmins in patients with these conditions, which could translate towards their active involvement in disease progression [11,12]. Similarly, alarmins were recently found to regulate inflammation and the behavior of immune cells implicated in the progression of asthma [13]. Other alarmins, such as ATP, are involved in the pathogenesis of asthma by mediating the release of other classes of alarmins [14]. The aim of this review is to discuss the potential involvement of selected alarmins in the pathogenesis of asthma.

## 2. Pathogenesis and Pathophysiology of Asthma

Asthma is a disease with complex pathophysiology. Several simultaneous processes synergize to translate into the clinical manifestation of asthma with a variety of symptoms. These processes involve immune and airway hyper-responsiveness, airway remodeling and airway smooth muscle hypertrophy, which leads to airway edema [1]. Firstly, the pathogenesis of asthma is strongly associated with the T2 inflammatory responses. These processes mainly involve group 2 innate lymphoid cells (ILC2s), Th2 cells, mast cells, eosinophils, basophils, IgE-producing cells, and B cells. Moreover, interleukin (IL)-4, IL-5, and IL-13 represent key cytokines implicated in the progression of asthma [15]. The vast majority of asthma patients demonstrate the T2-high-profile [6]. Additionally, patients with severe asthma also demonstrate a tendency towards the T2-associated comorbidities, such as atopic dermatitis, chronic rhinosinusitis, and allergic rhinitis. Interestingly, a recent report demonstrated that the presence of some T2 comorbidities could be used to predict response to biologics [16]. Eosinophils are one of the primary cellular components involved in the pathological hallmarks of asthma. They are involved in the processes of airway inflammation, goblet cell hyperplasia and mucus production [17,18]. Furthermore, these cells secrete eosinophil extracellular traps (EETs), structures that contain granule proteins and DNA fibers. EETs are considered to contribute to the eosinophilic inflammation present in asthma. Importantly, a greater presence of EET^+^ eosinophils was detected in patients with asthma than in controls. Moreover, an even greater difference was observed in patients with a severe course of the disease. The introduction of EET^+^ eosinophils in mice was associated with enhanced pro-inflammatory eosinophilic responses and enhancement of ILC2 cells [19]. By targeting IL-5, current biological therapeutics aim to suppress the activity of eosinophils and suppress asthma. Nevertheless, a recent investigation shed more light on the IL-based regulation of eosinophil functionality that is relevant to asthma. Specifically, IL-4 was found to regulate eosinophil lung recruitment, while IL-5 controls global maturation of eosinophils [20].

Th2 cells are central players in the T2 type of asthma, as they release a significant amount of T2 cytokines [21]. Furthermore, like other types of cells, Th2 lymphocytes secrete extracellular vesicles (EVs) that resemble properties of their source cells. Th2-derived EVs promote mast cell degranulation and eosinophil survival, key elements that contribute to the pathophysiology of asthma [22]. The importance of the Th2 cells in asthma was recently visualized in a study by Kumagai et al. [23]. Th2 differentiation is strongly linked to the expression of GATA3. Meanwhile, GATA3 expression demonstrates a relationship with polymorphisms within the region known as hG900. Enhancers located within this region promote the expression of GATA3. Mice lacking this region demonstrated reduced levels of eosinophils within the BALF and the lung. Moreover, there was a decrease in CD4^+^ T cells. Accordingly, the GATA3 expression is significantly reduced in murine CD4^+^ cells. These findings could imply that suppression of enhancers of Th2 differentiation could be beneficial in asthma. Nevertheless, the authors also demonstrated an increase in neutrophil infiltration and IL-17.

ILC2 represents the population of cells strongly implicated in asthma. Upon stimulation, they produce IL-5 and IL-13, thus contributing to the progression of changes in asthma [24]. IL-13 is a type 2 cytokine that plays a role in eosinophilic disorders. In the respiratory system, IL-13 enhances airway hyperreactiveness and mucus production, thus contributing to the pathogenesis of asthma hallmarks. Consequently, targeting IL-13 is being considered as a potential therapeutic target in asthma, with several clinical trials being performed so far [25].

**Figure 1:**
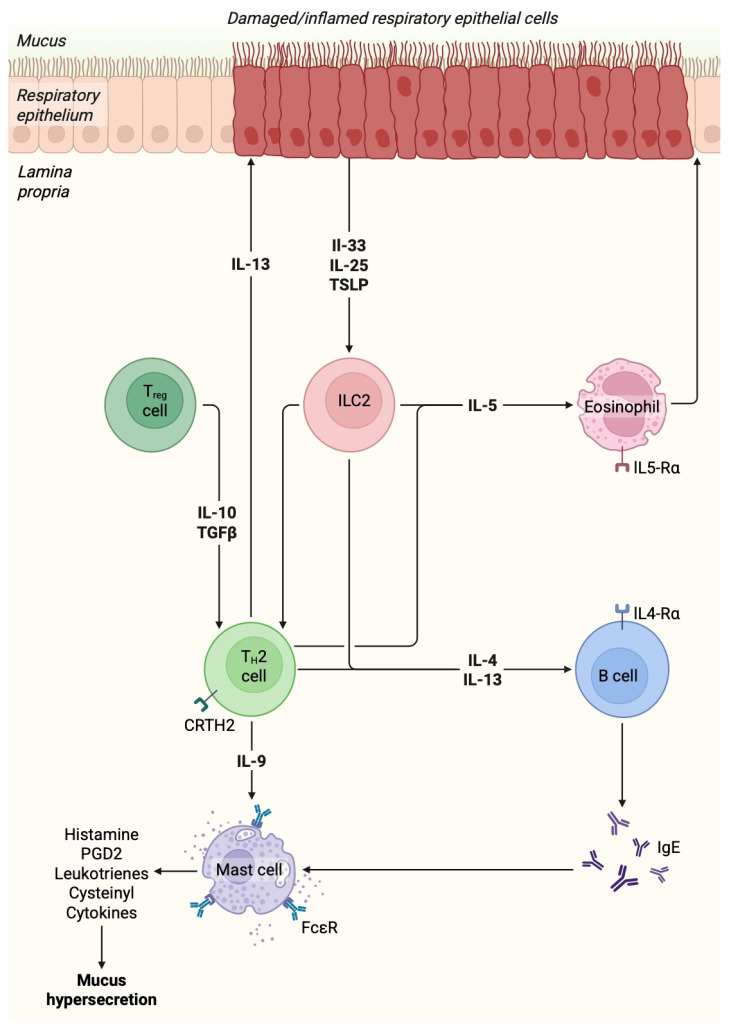
Inflammatory components involved in the pathogenesis of asthma. Created in BioRender. Physiology, D. (2025) https://BioRender.com/gxxqrlc.

Therefore, the T2 asthma is a complex disease associated with the presence and involvement of several various cells (Figure 1). Nevertheless, there is a group of asthmatic disorders that are not related to atopic responses or allergy. These conditions are known as non-T2 asthma and may result from alterations in gut microbiome, viral infections, environmental factors, as well as obesity [26].

## 3. Alarmins and Asthma

### 3.1. High-Mobility Group Box-1

This protein was first extracted from the chromatin of the calf thymus during ion exchange chromatography studies in the 1970s by Goodwin and Johns. As a result of the conducted research, which included electrophoresis in a polyacrylamide gel, analysis of amino acids, including N-terminal amino acids, and on the basis of molecular weights, these molecules were characterized as proteins of high mobility—HMG [27]. Currently, HMG is a family of proteins that includes three subfamilies: HMGB, HMGN and HMGA. Each of these groups is characterized as highly conserved nuclear proteins [28]. HMGB1 is a non-histone nuclear protein with DNA-binding domains. It is encoded by the *HMGB1* (13q12) gene [28,29,30]. The mass of the HMBG1 molecule is determined at the level of 24,894 Da. It consists of 215 amino acids. Structurally, GMGB1 can be divided into three parts: A-box, B-box, and acidic tall [28,29,31]. The B-box is called the “functional region of inflammation” made up of about 74 amino acids and is mainly responsible for binding to TLR4 and RAGE receptors, which are associated with the regulation of the production of pro-inflammatory cytokines, i.e., IL-4, IL-6 and TNFα, which is why HMGB1 is associated with many inflammatory diseases [30,31]. The antagonist of the B-box area is the A-box, also known as the “anti-inflammation domain”, made up of more than 70 amino acids. Both of these domains have the ability to bind to DNA, the ability of which is further enhanced at the time of acidic tall detachment [32,33]. The acidic tall region itself, which is negatively charged, is responsible for the stabilization of HMGB1 and enhances the ability to bend DNA. In addition, it intensifies the anti-inflammatory activities of the A-box [30]. HMGB1 is expressed in many human cells and tissues [28,29]. This protein possesses two nuclear localization sequences that are identified by the intracellular transport team. Intracellular HMGB1 primarily occurs in the cell nucleus, where it binds to chromatin in its physiological state. In addition, it is responsible for regulating replication, gene transcription, but also cell polarization and maturation, and maintaining telomere stability [28,34]. In states of excessive cellular stress, HMGB1 enters the cytoplasm and then into the extracellular space. This can occur as a result of cellular necrosis or dynamic secretion by immune cells, i.e., DC, NK cells and macrophages, in response to an inflammatory response [28,29,35]. Cytoplasmic HMGB1 has the ability to bind the Beclin-1 protein, resulting in the initiation of mitochondrial autophagy [36]. However, when released into the extracellular space, this protein is converted into an inflammatory factor that stimulates inflammatory reactions and is involved in many different immune responses. HMGB1 is a pro-inflammatory mediator during infection and tissue damage, leading to the release of large amounts of TNFα, IL-1, IL-6, and IL-18 [29,37,38]. Interestingly, this protein can stimulate the production of cytokines by monocytes, also affecting the ability of macrophages to phagocytic particles. HMGB1 also contributes significantly to the migration of neutrophils, monocytes, and dendritic cells [28,29,38,39]. It has also been shown that HMGB1 can form a complex with almost any type of nucleic acid and LPS (lipopolysaccharides), leading to an increased immune response [38]. In the case of the adaptive response, HMGB1 is responsible for the activation of APC cells through their migration and maturation. As a consequence, T cells differentiate and proliferate both autocrinally and paracrinally, which then release a wide range of different cytokines [28,29].

HMGB1 can induce multiple signaling pathways as a result of integration with multiple receptors. This protein can interact with RAGE. It is a receptor associated with the recognition of patterns that are involved in distinguishing endogenous particles formed during cell death or defects as a result of infection, physiological stress or chronic inflammation [40]. RAGE is expressed on the surface of many innate response cells, i.e., monocytes, macrophages, and DC cells [41]. Extracellular HMGB1 can activate RAGE, resulting in activation of the NF-κB pathway and the MAPK pathway, and thus leads to the production of TNFα and IL-8 by neutrophils [41,42,43]. RAGE-based signaling also affects the regulation of inflammation and the differentiation, migration, and even cell death [41]. HMGB1 also has the ability to bind to TLRs, primarily TLR2 and TLR4. As in the case of RAGE, the receptors are located on the surface of DC cells, macrophages and neutrophils. TLR stimulation leads to the proliferation and migration of neutrophils and monocytes, resulting in the release of a large number of cytokines. This is followed by the activation of agglutination, which leads to the maturation and acquisition of adhesion capacity by DC cells, and then to the release of cytokines and pro-inflammatory mediators [44]. HMGB1 can also induce the JAK/STAT pathway, which leads to the release of TNFα, IL-1, and IFN-γ [38].

The level of HMGB1 in sputum, plasma, and serum in people with asthma is significantly elevated compared to people with healthy asthma. Also, the level of this protein in people with severe asthma is higher than in patients suffering from mild or moderate forms of the disease. Thus, HMGB1 levels are significantly positively correlated with disease severity. In addition, HMGB1 is inversely correlated with forced expiratory volume in 1 s (FEV1), thus suggesting the involvement in respiratory restriction [38,43,45,46]. Therefore, HMGB1 could serve as a biomarker of asthma severity [38,43,45].

The detailed mechanism of action of HMGB1 in the pathophysiology of asthma is not yet well understood. However, the correlation of the level of this protein with the severity of the disease has led many scientists to try to explain the molecular mechanisms of HMGB1 in many experimental models. HMGB1 acts on Alveolar epithelial type II cells (AT2) and plays a very important role in the regulation of chronic inflammation of the respiratory tract and remodeling in the mouse model of asthma caused by allergies. It should be borne in mind that AT2 cells, which are the so-called progenitor cells, have the ability to secrete surfactants as a consequence of lung damage [47]. Another study showed that the expression of HMGB1 in patients was positively correlated with the occurrence of sputum eosinophilia and with the expression of IL-5 and IL-13 in the same material. Therefore, HMGB1 is referred to as an eosinophilic chemoattractant [48]. Due to the fact that eosinophils prefer necrotic areas, it has been shown that it is the necrotic cells located within the epithelium of the respiratory tract that stimulate eosinophils. Added to this is the action of HMGB1, which affects the survival of eosinophils, which is a considerable problem for mild asthmatics [49].

The previously mentioned pathways associated with RAGE, TLR4, NF-κB, JAK/STAT, but also TGF-β, are thought to play an important role in the pathogenesis of asthma. Each of them triggers inflammatory reactions leading to cell damage. RAGE-based signaling is likely to exacerbate disease severity [41,50]. HMGB1/RAGE signaling affects the bronchial modifications and epithelial-to-mesenchymal transition (EMT) stimulated by TGF-β1. It is associated with differentiation and maturation of Th17 lymphocytes and the release of IL-17, thus leading to severe steroid-resistant asthma [51]. Another study noted that HMGB1/RAGE interaction had an effect on the increased expression of TNFα, thymic stromal lymphopoietin (TSLP), matrix metalloproteinase 9 (MMP-9) and vascular endothelial growth factor (VEGF) [52]. Each of the released cytokines has a significant impact on the development of asthma. TNFα supports the recruitment of eosinophils and neutrophils to the respiratory tract. This is related to its effect on the endothelium, which enhances inflammatory responses [53].

On the other hand, the presence of a significant concentration of VEGF corresponds to the degree of narrowing of the airways and the presence of blood vessel permeability, which results in thickening of the mucous membrane of the respiratory tract walls and intensification of the progression of asthma symptoms [54]. TSLP contributes significantly to inflammation of the respiratory tract [55]. MMP-9, on the other hand, is characterized as an important element contributing to the infiltration of eosinophilia in the respiratory tract and to the airway remodeling [35]. Interestingly, the concentration of HGMB1 and RAGE in sputum samples of asthmatics is positively correlated with the percentage of neutrophils and their activity compared to controls [56]. HGMB1 is an ideal chemoattractant for neutrophils and inhibits their apoptosis [57]. Neutrophilic airway inflammation is associated with long-term restriction in air exchange in severe asthma [43]. Additional stimulation of the release of TNFα, IL-6, and IL-8 from monocytes further promotes the induction of neutrophilic asthma [58].

Several studies have been conducted that investigated the effects of different TLR-related pathways on the pathogenesis and course of asthma. In an experiment conducted on mice with an HMGB1-conditioned knockout, it was shown that HMGB1/TLR4 contributes to the production of IL-1β in isolated stretched alveolar macrophages, but also to other cytokines, leading to respiratory tissue damage [47]. TLR4 activity is largely dependent on IL-33, particularly in allergic inflammation. This was proven in an experiment conducted on MLE-12 (mouse lung epithelial cells). In humans, TLR4 is naturally expressed in lung epithelial cells and is responsible for signaling epithelial and endothelial damage, as well as apoptosis and necrosis [59,60].

The JAK/STAT pathway, which is associated with HMGB1, is primarily responsible for the release of TNFα, IL-1 and IFNγ. Moreover, the JAK1/STAT3 pathway mainly promotes the polarization of naïve CD4^+^ T cells by phosphorylation to Th17. In turn, they release IL-17, which induces neutrophil inflammation in the respiratory tract, especially in non-allergic asthma [38]. In addition, HMGB1 stimulates DC activation and polarization of naïve helper cells towards Th17. This protein also affects Th2, which significantly contributes to the increase in IL-4 secretion, which in turn affects B lymphocytes in the production of IgG and IgE [41]. It has also been shown that HMGB1 is secreted specifically by bronchial epithelial cells, which only increases the release of cytokines [38].

**Figure 2:**
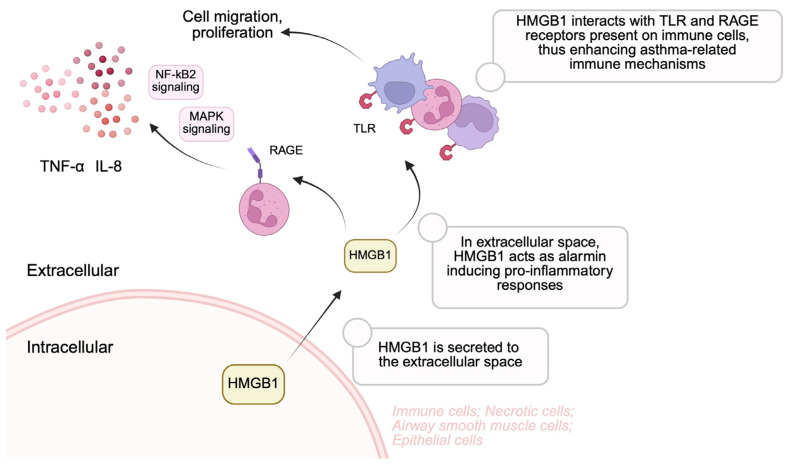
Released into the extracellular space, HMGB1 acts as an endogenous alarmin, stimulating pro-inflammatory responses. It interacts with TLR and RAGE receptors, thus stimulating inflammation. Created in BioRender. Kiełbowski, K. (2025) https://BioRender.com/n88j960.

There are several possible therapies that can reduce the concentration of HMGB1 protein. One of these is inhaled glucocorticoid therapy, which reduced the level of this protein in the sputum of children with moderate asthma [46]. In the case of studies conducted on mouse asthma models, it has been shown that the use of HMGB1 neutralizing antibody reduces the production of Th1, Th2, and Th17 cells, which has a significant effect on the weakening of respiratory tract inflammation or mucus formation [61]. In a mouse model of diisononyl phthalate (DINP)-induced asthma, lung tissue was analyzed along with bronchial alveolar lavage fluid, following anti-HMGB1 treatment, which inhibited HMGB1 expression and visibly reduced inflammatory infiltration in the airways. Analysis of CD4^+^ T cells located within the range of mediastinal lymph nodes and lung tissues showed a decrease in the number of Th1, Th2, and Th17 cells compared to the control sample [62]. In a study conducted on female BALB/c mice, it was shown that the use of anti-HMGB1 antibody had a positive effect on reducing the occurrence of inflammatory mediators, along with the accumulation of inflammatory cells and IgE (immunoglobulin type E). Reduced airway hyperreactivity, smooth muscle thickness and collagen levels in the lungs were observed. The introduction of IL-1β therapy additionally contributed to the increase in the expression and thus the production of TGF-β1, MMP-9, and VEGF [63]. When treating a mouse asthma model, TDI (toluene-2,4-diisocyanate) with EP (ethyl pyruvate) reduced neutrophil infiltration depending on the dose used [64]. The use of vitamin D in animal models led to a weakening of inflammation within the respiratory tract and to a reduced apoptosis of lung tissue. The underlying mechanism of action involved suppression of the HMHB1/TLR4/NF-κB axis [65]. Another study showed that the use of miR-15b-5p in mice significantly reduced the occurrence of peribronchial inflammatory cells, while leading to a reduction in inflammation and remodeling of the airways. In addition, overexpression of miR-15b-5p significantly led to inhibition of apoptosis and activation of HMGB1, which in turn affected TLR4 receptors [47]. A study conducted on human airway smooth muscle cells showed that the use of astragaloside IV (ASIV) to treat asthma significantly affects HMGB1 by weakening the expression of this protein and thus preventing its entry into the cytoplasm. AS-IV also blocks signaling between HMGB1 and RAGE and leads to a reduction in lung inflammation in mice [42]. Figure 2 demonstrates the pro-inflammatory activity of HMGB1 when it is released into the extracellular space.

### 3.2. S100

The S100 protein was first described as a result of studies that made it possible to determine proteins that fully dissolve in ammonium sulfate [66]. Today, the term refers to 25 proteins, with 16 S100A proteins (S100A1–S1000A16) and variations such as S100B, S100G, etc. [67,68]. Each molecule belonging to this group of proteins shows a similar molecular weight, manifested in the range of 10–12 kDa. It is noteworthy that these proteins present a 25–65% similarity in the amino acid sequence [69]. Each protein belonging to the S100 protein group is encoded by a different gene, of which as many as 16 genes are closely related to the 1q21 region on chromosome 1 [70]. The basic building block of the S100 protein is the protein monomer, which is made up of structural parts, i.e., helix–loop–helix, which are also referred to as EF-hands [69,71,72]. The 2 EF-hands are integrated into the hinge surface and are surrounded by conservative hydrophobic residues that are located at the N- and C-ends [69]. To achieve full activation of the S100 protein, they bind to transition metals—Ca^2+^, Mn^2+^, Zn^2+^, Cu^2+^. Subsequently, they must achieve the conformation of homo- or heterodimers [73,74]. For this reason, different varieties of the S100 protein are characterized by a very similar structure. In addition to the apparent variation within the C-terminal extension and the hinge, there is a definite sequence difference between the S100 proteins, which makes it possible for differences in function to occur [71]. Therefore, these proteins are expressed in various cells and tissues in the human body. They can be found both in the epithelium of the respiratory tract, gastrointestinal tract, urinary tract, but also in immune cells, i.e., macrophages, monocytes, neutrophils, DC cells and lymphocytes [69]. S100 proteins have various functions both inside and outside the cell. In the former, they are mainly responsible for monitoring the function of the immune system, so they are related to the regulation of transcription, growth, division, and differentiation of cells. They are also responsible for the control of cell shrinkage and motility. In addition, it has been shown that these proteins are involved in maintaining the proper cell structure and dynamics of cytoskeletal components. Furthermore, S100 proteins are associated with protecting the cell from oxidative damage. All these activities are possible due to the interaction of S100 proteins with various effector proteins and binding to various transition metals [72,75]. When bound to Ca^2+^ ions, S100 proteins function as sensor proteins, resulting in a change in the conformation of the structure. This leads to interactions with various target proteins, thereby affecting their activity. Unfortunately, the interaction with Zn^2+^ and Cu^2+^ ions is unknown [76]. If the S100 protein enters the extracellular space, it can have an autocrine or paracrine effect on mechanisms associated with inflammation [72]. Release can occur as a result of cell necrosis or through cell excitation, which can take place, for example, as a result of changes in the extracellular potential of Ca^2+^ and K^+^ [67]. Extracellular S100 proteins can interact with RAGE, TLR, and G protein-coupled receptors (GPCRs).

In the first two receptors, S100 proteins act as cytokines that, after binding to receptors, contribute to the initiation of a signaling cascade leading to the recruitment, proliferation, and differentiation of immune cells [71]. They can also turn on pathways related to MAPK and NF-κB, affecting the production of pro-inflammatory mediators. S100A8 and S100A9 have the ability to interact with the TLR4 receptor. S100A7, S100A12, S100A8, S100A9, and S100B bind to RAGE [67]. The immunoregulatory properties of S100 proteins are complex. Members of the S100 family are expressed by macrophages, which can be enhanced due to the activity of cytokines. In turn, their greater expression can further promote pro-inflammatory responses [71].

Furthermore, extracellular expression of S100A8/A9 has been shown to activate neutrophils. The phosphorylated form of the S100A8/A9 induces pro-inflammatory cytokines expression, thus moderating the pro-inflammatory function of neutrophils [77]. These molecules are particularly related to neutrophils. Sprenkeler et al. [78] demonstrated that S100A8/A9 is released from neutrophils through the process of neutrophil extracellular trap secretion, also known as NETosis. S100 proteins take part in leukocyte migration through activation of MAPK and NF-κB pathways, and recruitment of monocytes by activating the RAGE-dependent NF-κB [69]. Therefore, these interactions between the S100 proteins and neutrophils could indicate the involvement of these alarmins in the pathogenesis of neutrophilic asthma.

A number of S100 proteins are involved in the pathogenesis of asthma. A study compared serum levels of S100A9 in asthmatic and healthy patients. This protein is released by activated neutrophils; S100A9 levels were elevated in samples from patients suffering from neutrophilic asthma than in the ones from healthy individuals, thus presenting a potential for this protein as a biomarker for asthma. Moreover, these results correlated with sputum neutrophil count and a higher prevalence of asthma with more severe symptoms. These findings demonstrate that S100A9 is a mediator playing a key role in regulating airway inflammation and activation of the immune cells [79]. A study focused on S100A4 protein and its connection to airway inflammation, epithelial barrier dysfunction and release of T_H_2 by VEGFA/VEGFR2 pathway, showing that increased secretion of this alarmin is related to the development of asthma [80]. The same protein has been linked to inducing mast cell activation and oxidative stress stimulation via the RAGE and PPAR-γ pathways [81], as well as mediating inflammation in airway smooth muscles [82]. By inhibiting S100A4 expression with miR-124-3p, researchers were able to reduce inflammation in asthmatic mouse models [83]. Experiments involving S100A4^−/−^ mice have shown that those animals displayed less severe asthma signs, further supporting the role of S100A4 in regulating mast cell activation [84]. Interestingly, S100A11 seems to relieve airway hyperresponsiveness through relaxing airway smooth muscles, which can potentially become a therapeutic target [85]. Two varieties of the S100 protein—S100A8 and S100A9—can form a heterodimer, also known as calprotectin. The formation of this structure is possible thanks to the activated inflammatory cells, which primarily include neutrophils. Their increased activity is found in the sputum of patients with asthma, which translates into the presence of the S100A8/S100A9 complex, which is largely correlated with the severity of the disease [86,87]. A positive correlation was also observed between the S100A8/S100A9 complex and monocytes in the respiratory tract, the mucous membrane of the respiratory tract, but also in mucus plugs [88]. The increased expression of S100A8 was demonstrated in peripheral blood mononuclear cells at the time of asthma exacerbation, while additional S100A9 was elevated in neutrophils and macrophages in patients with untreated asthma [87,89]. Interestingly, the expression of S100A9 and RAGE was elevated in circulating fibroblasts in asthmatics along with acute exacerbations and chronic obstructive asthma in comparison with asthmatic patients with normal lung function [90]. Elevated levels of S100A12 in the sputum, mucosa, and mucus plugs of patients with eosinophilic asthma have also been observed compared to other types of asthma and controls [91].

Venestatin, produced by *Strongyloides venezuelensis,* has therapeutic potential in asthma due to its antagonistic function towards RAGE-mediated pathways, including those stimulated by S100 proteins [92]. Dapagliflozin, a long-known drug used in the treatment of diabetes, reduces the S100A4 levels, leading to mitigated bronchospasm. The bronchodilator and anti-inflammatory properties of this substance could lead to a new alternative for asthma therapy [93]. Paquinimod, a quinoline-3-carboxamid inhibiting S100A9, decreases the number of inflammatory cells in animal models; thus, it should be investigated further in efforts to find a novel asthma treatment [94]. However, in the case of studies conducted on a mouse model of acute asthma caused by ovalbumin, (associated with an increase in the expression of S100A4), treatment with an anti-S100A4 antibody was applied. During the therapy, inflammation of the airways was inhibited as a result of a decrease in the accumulation of inflammatory cells, inflammatory mediators, as well as hyperreactivity of the airways [95]. In a mouse model of neutrophilic asthma OVA/CFA (ovalbumin/complete Freund’s adjuvant), an experiment was performed using an anti-S100A9 antibody, which resulted in a restoration of normal neutrophil counts, as well as a reduction in the levels of Th1, Th17, as well as IL-1β, IFN-γ, and IL-17 in mouse lung lysates. In addition, a reduction in airway reactivity was observed [87].

Overall, several proteins from the S100 family have been studied with the object of better understanding the pathogenesis of asthma and finding new potential treatment methods. Researchers not only found links between pathways induced by those proteins and asthma symptoms but are also working on several new substances moderating these reactions in order to find better therapies for patients suffering from this disease. S100 should be further studied as a biomarker of asthma and therapeutic targets.

### 3.3. Interleukin-33

IL-33 is an alarmin that belongs to the IL-1 family. Alongside other members of this group, it plays a significant role in modulating immune system responses. It consists of two domains: N-terminal chromatin-binding domain and C-terminal domain, which exhibits cytokine activity, separated by a central segment [96]. After binding with its receptor ST2, IL-33 activates several pathways, including NF-κB and MAPK. Moreover, it promotes IL-5 and IL-13 production by T_H_2 cells [97]. In CD8^+^ T cells, IL-33 promotes metabolic activity by enhancing the expression of GLUT1 and enzymes involved in glycolysis. Moreover, it plays an important role in stimulating CD8^+^ T cells’ activity [98] and stem-like features in viral infections [99]. The alarmin mediates inflammatory responses in acute respiratory disease syndrome (ARDS) by regulating the presence of NK cells [100]. Additionally, IL-33 also influences macrophage polarization, as it was found to synergize with IL-4 to enhance the alternatively activated phenotype of these cells [101]. Thus, the cytokine has profound immunoregulatory properties, as demonstrated by studying its diverse influence on immune cells.

Several studies have analyzed the IL-33 association with asthma. Single-nucleotide polymorphisms (SNPs) of IL-33 have been proven to be strongly connected to this chronic disease. Elevated levels of this alarmin were found in samples of bronchial biopsies and sputum of patients suffering from this condition. A particularly strong association has been reported with eosinophilic asthma [102]. Indirectly, IL-33 can point to the severity of asthma. Specifically, organisms can produce endogenous antibodies directed at cytokines. Levels of anti-IL-33 antibody are greater in patients with asthma compared to healthy volunteers. Additionally, their concentrations are higher in the serum of patients with severe asthma than in patients with a moderate condition [103]. A study by Poulsen et al. proved that IL-33 present in airways moderates the function of type-2 cytokines, which suggests that this interleukin is enhancing the inflammation induced by those particles in acute asthma [104]. A recent study found that inflammation of the airway was decreased in IL-33^−/−^ asthma mouse models [105], further supporting the hypothesis that this particular alarmin is connected to the pathogenesis of asthma. Studies have demonstrated a distinct connection between elevated levels of IL-33 and an allergic type of asthma. Basophils activated by this molecule regulate T_H_2 lung tissue entry, which directly boosts airway inflammation [106]. Clearly, IL-33 is involved in many asthma pathogenesis pathways, and its secretion leads to an increase in inflammation, which is responsible for the onset and worsening of the condition.

IL-33 involvement in asthma progression has the potential for discovering a new treatment involving inhibiting its secretion and analyzing the treatments known to medicine today. Recently, there have been several studies focused on IL-33 specifically. Wu et al. examined 265 children suffering from asthma and discovered that IL33-IL1RL1-IL1RAP complex polymorphisms may affect not only the pathogenesis of asthma itself, but also subsequent inhaled corticosteroid (ICS) response. They found that children with the IL33 rs4742170 genotype had a greater risk of poor response to treatment involving ICS [107]. An analysis of IL-33 stimulated signatures in the samples of sputum collected from severe neutrophilic asthmatics highlights the possible potential of IL-33 targeted treatments [108]. The previously mentioned immunoregulatory features of IL-33 are thought to significantly contribute to the underlying processes being studied in asthma. As the alarmin was found to regulate the activity of CD8^+^ T cells in the context of viral responses, it was also observed that the cytokine can promote T2-type immune responses in Tc cells. Importantly, a greater presence of Tc2 cells (Tc cells producing type 2 mediators) is associated with asthma exacerbations [109], thus highlighting a potential role of IL-33 in these episodes. Poulsen et al. also emphasized the possibility of a novel therapy that could be achieved by blocking IL-33 [104].

Researchers have already tried to discover a drug that would inhibit IL-33. A trial conducted by Wechsler et al. analyzed an itepekimab used in monotherapy. It is a human IgG4P monoclonal antibody targeted specifically at IL-33. Results demonstrated that itepekimab improves lung function and reduces the chances of a loss of asthma control [110]. Another trial investigated astegolimab—a human IgG_2_ monoclonal antibody designed to inhibit ST2, an IL-33 receptor. A study demonstrated promising results, as astegolimab reduced the asthma exacerbation rate in patients suffering from low blood eosinophil asthma [111]. Interestingly, widely used and long-known drugs could be repurposed for the treatment of asthma. For example, recent experiments studied the potential role of metformin in mice models of asthma. Investigations demonstrated that metformin reduced the presence of immune cells stimulated by IL-33 in BALF and lungs. Moreover, the addition of metformin to IL-33 reduced the presence of IL-13 and IL-5, thus suggesting the relationship between the long-known biguanide and alarmins in asthma [112]. Overall, IL-33, as well as its receptors and complexes, has the potential to become a biomarker associated with asthma development and also treatment response. Further studies and continuation of clinical trials are needed to determine whether drugs inhibiting pathways induced by this alarmin are going to be used in the treatment of asthma.

### 3.4. TSLP

Thymic stromal lymphopoietin (TSLP) was first recognized in 1994 as a cytokine secreted by thymic stromal cells, which contributed to lymphopoiesis B in vitro [113]. Today, it is known that TSLP is expressed within the epithelial cells of the lungs, intestine, in Hassall’s corpuscles in the thymic medulla, lymphoid tissues, and even in smooth muscle cells or lung fibroblasts [114]. The gene for TSLP is located on chromosome 5q22, in close proximity to genes encoding IL-4, IL-5, IL-9, and IL-13. This cytokine exists in a four-helix form, which is stabilized by three disulfide bridges. They are composed of four α-helices, which are referred to in the nomenclature by the designations from αA to αD. Each of them is connected by a BC loop along with two elongated areas of the AB and CD loops [115,116,117]. In addition, two isoforms of TSLP can be distinguished—a short one consisting of 60 amino acids and a long one with 160 amino acids in its structure. Both of them are characterized by the presence of specific gene promoters that are regulated differently by external factors [118].

In the case of the shorter isoform, expression occurs in many tissues in a constitutive manner, and its role is mainly related to the maintenance of homeostasis. The expression of the longer isoform is induced and leads to the initiation of inflammatory responses [119]. Its level is mainly upregulated by molecules such as IFNγ and TNFα or IL-1β, IL-4 and IL-13 [120]. TSLP can promote a type 2 immune response that directly targets immune cells such as T cells, NK cells, and mast cells. This process can also occur indirectly as a result of the stimulation of CD11c^+^ NK cells, which, after entering the lymph nodes, stimulate the polarization of CD4^+^ T cells towards type 2 cells and initiate complete proliferation as a result of the response to the antigen. This entire reaction leads to the release of IL-4, IL-5, IL-13, among others [116,121,122]. These interleukins, in turn, affect the differentiation of B cells, which then secrete significant amounts of IgE [123]. As a result of the interaction of TSLP with IL-1 and TNFα, the production of Th2 cytokines by mast cells may be induced [114,124]. TSLP also mediates the initiation of maturation and proliferation of DC cells and naïve T cells. It is also possible to trigger a cascade of reactions leading to the release of chemokines [116].

First of all, the interaction of TSLP with CD34^+^ has a positive effect on the transition towards the post-inflammatory phenotype associated with type 2 cytokines, but also towards the proliferative phenotype responsible for the activation of both basophils and eosinophils [125,126]. The influence of TSLP on basophils is not fully understood, but it is known that a set of DC-T cells, together with basophils, are responsible for the type 2 immune response. As a result of DC cell activation, naïve T cells are promoted with OX40L, which in turn affects the release of IL-3 that supports the recruitment of basophils and the production of IL-4 [116,127]. In the case of eosinophilia, it has been shown that TSLP significantly slows down their apoptosis. In addition, this alarmin is responsible for controlling the expression of ICAM-1 and CD18 but also enhances the adhesion of eosinophilia to fibronectin. Additionally, it is responsible for the release of IL-6 and chemokines—CXCL1, CXCL-2, CXCL-3 [116].

**Figure 3:**
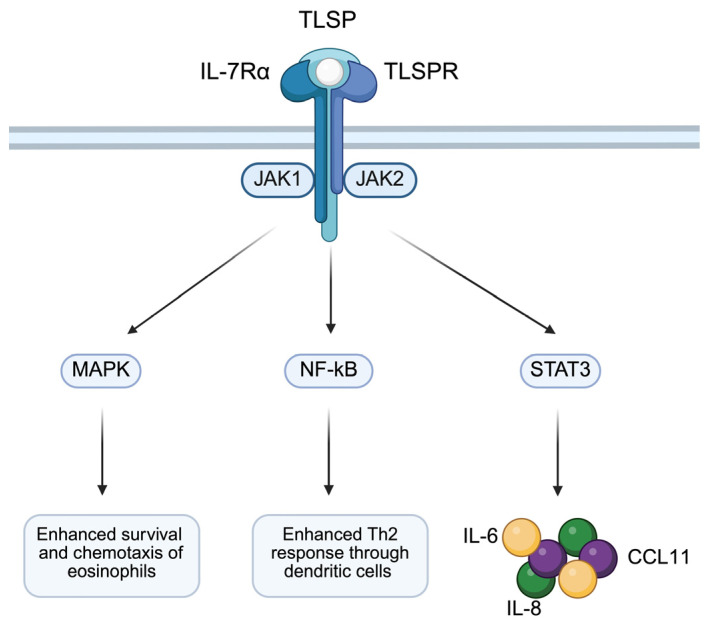
The binding of TSLP with its receptor stimulates a signaling pathway that mediates inflammatory responses observed in asthma. Created in BioRender. Physiology, D. (2025) https://BioRender.com/v70d135.

TSLP is able to initiate intracellular type 2 signaling by binding to a set of heterodimeric receptors. Among them, a specific negatively charged TSLP receptor (TSLPR) can be distinguished. It has almost a 24% similarity with the γ chain of the receptor common to IL-2, IL-4, IL-9 and IL-15, but it does not have the δ chain shared by IL-2 and IL-7Rα [128]. As a result of the uptake and modification of TSLP under the influence of TSLPR, the next stage is associated with the involvement of IL-7Rα to initiate intracellular pro-inflammatory pathways such as the JAK/STAT cascade [116,117]. JAK1/JAK2 are responsible for phosphorylation and transcription of STAT1/3/5, as well as MAPK and NF-κB, depending on the type of cell. As a result of STAT3 activation, IL-6, IL-8 and CCL11 are released, which leads to the initiation of cell proliferation, anti-apoptotic activity, and DC cell migration. Activation of the MAPKs pathway contributes to the survival and chemotaxis of eosinophilia, while the NF-κB pathway increases the Th2 response with the help of DC cells [129,130] (Figure 3).

Scientists have been trying to define the role of TSLP in the pathogenesis of asthma. Levels of TSLP are increased in patients with asthma [131], while higher concentrations were correlated with a more severe condition [132]. This cytokine was found in the cytoplasm of human lung macrophages. Researchers found that both T2-high and T2-low stimulation lead to the release of this molecule [133]. A study by Murrison et al. demonstrated that genetic variants contributing to the elevated expression of TSLP are associated with a greater risk of asthma [134]. Interestingly, there is also a negative correlation between the amount of epithelial and submucosal cells (which were previously expressing TSLP) with FEV1 [55]. A correlation related to airway hyperreactivity was also demonstrated with serum IgE concentration and the number of TSLP-expressing mast cells [135]. Moreover, IL-4, which is mainly responsible for increasing permeability within the epithelial cells of the respiratory tract, also causes an increase in the level of TSLP [136]. In addition, another study showed that increased expression of the TSLP receptor located on the surface of alveolar macrophages correlates with a prolonged course of the disease and reduced lung function [137]. Toki et al. found that TSLP and IL-33 enhanced each other’s expression in the lung tissue, further enhancing type-2 inflammatory response [138].

In severe asthma, TSLP is referred to as a molecule that plays the most important role in triggering the cascade of the inflammatory response [116]. In the case of paucigranulocytic asthma, TSLP-mediated cross-reactions occur between mast cells and the building cells of the respiratory tract, i.e., fibroblasts, smooth muscle cells, or epithelial cells [139].

Therapeutics targeting TSLP have already been designed. Their use is being studied in patients with asthma. One of the promising substances seems to be tezepelumab. It is an anti-TSLP monoclonal antibody, which essentially blocks this molecule from interacting with its respective receptor. Tezepelumab has been proven to reduce IL-33 and T2 cytokines levels by blocking TSLP, which reduced the inflammatory response of the airways [140]. Another study has discovered that this antibody reduced the levels of not only TSLP but also other biomarkers, such as eosinophils, IL-5, and IL-13 [141]. Tezepelumab treatment has also been associated with reducing airway hyperresponsiveness to mannitol and reported no concerning safety findings [142]. The most recent publications further confirm the efficacy and safety of the therapeutic [143,144,145]. Importantly, treatment with tezepelumab provides psychological benefits as it improves the perception of asthma triggers, which translates into improved quality of life [146].

Ecleralimab is another TSLP-neutralizing medication. It significantly reduces airway inflammation and bronchospasm induced by allergens in patients with mild atopic asthma, demonstrating a manageable safety profile [147]. Lunsekimig is a treatment suppressing the activity of both IL-13 and TSLP. Researchers conducting the first human study found it to be well tolerated [148]. Lastly, inhibiting ferroptosis can also be a new way of treating neutrophilic asthma. Researchers studied lipoxstatin-1 (Lip-1) and found it to be reducing the expression of TSLP along with other biomarkers, leading to alleviation of neutrophilic asthma [149]. In an experiment involving cynomolgus monkeys, previously obtained mABs directed against TSLP were introduced. After 6 weeks of examination in BALF, a decrease in eosinophil counts and IL-13 levels was noted. In addition, there was a decrease in airway resistance [150]. The use of bispecific antibodies represents another potential approach in targeting cytokines and inflammatory mediators. CDX-622 is a bispecific antibody that targets stem cell factor and TSLP and was recently studied in cynomolgus macaques. Perhaps, such therapeutics will be tested in the context of asthma in the future [151]. Lunsekimig is a bispecific drug that targets TSLP and IL-13. The results of a phase 1b clinical trial showed that it reduced the levels of exhaled nitric oxide fraction and type 2 inflammation biomarkers in the blood in patients with asthma [152].

Another group of potential therapeutics represent natural agents. The use of baicalin, a flavonoid derived from *Scutellaria baicalensis* Georgi, has been evaluated in ovalbumin-stimulated mice. The results demonstrated that there was a marked decrease in eosinophils in BALF, cytokines, and serum IgE [153].

Overall, current evidence demonstrates that TSLP is strongly associated with the pathogenesis of asthma, driving and enhancing the inflammatory response through multiple targets.

### 3.5. Interleukin-25

IL-25 is another important cytokine implicated in the pathogenesis of asthma. It belongs to the IL-17 family, and it is also known as IL-17E. The activity of the molecule is linked with allergic conditions. Specifically, it regulates the type 2 immune responses. Among IL-25-expressing cells, there are immune cells and lung epithelial cells. Therefore, it has been suggested that the cytokine could be involved in pulmonary responses to allergens [154]. IL-25 has been investigated in the context of asthma quite extensively. Furthermore, it has been suggested as a potential therapeutic target as it was associated with the inflammatory responses. Yao et al. [155] studied IL-25 in asthma-related fixed airflow limitation (FAL). Researchers observed a greater presence of circulating fibroblasts expressing ILRB (IL-25 receptor) in patients with FAL than in asthmatic patients without this airflow limitation. In animal models, the authors demonstrated that IL-25 stimulation promotes the presence of ILRB^+^ fibroblasts in lung parenchyma and in the peribronchial regions. Additionally, the cytokine enhanced pro-fibrotic signalling and responses. Further investigations proved that IL-25 links eosinophils with T2 immune responses, as stimulation of eosinophils and naïve T cells in co-culture with IL-25 led to the greater abundance of CD4^+^ IL-4^+^ Th2 cells. By contrast, there were no such observations when T cells were cultured without the presence of eosinophils [156].

Recent studies also demonstrated a broader picture regarding the network of interactions involving IL-25. It is widely known that the expression of numerous molecules is regulated by the activity of microRNA (miRNA), a class of non-coding RNA (ncRNA). Inflammatory responses driving the progression of asthmatic changes involve the release of extracellular ATP, another alarmin that stimulates the expression of IL-25. In patients with type-2 high asthma, which is associated with enhanced type-2 immunity, there is a greater expression of miR-206, as compared to patients with type-2 low immunity. miR-206 downregulates CD39, an enzyme that catalyzes the degradation of ATP. Consequently, enhanced presence of miR-206 reduced degradation of extracellular ATP, thus leading to stimulation of IL-25 production [157]. Thus, IL-25 is implicated in a network of interactions involving other alarmins (ATP) and miRNA (miR-206), which mediate its effects on type-2 immunity.

Thus far, we have discussed recent studies that revealed the complex role of IL-25, which has been previously correlated with asthmatic airway inflammation. Another aspect that should be considered is whether IL-25 could become a therapeutic target or biomarker in asthma. An early study (2007) demonstrated that the use of IL-25 targeting monoclonal antibody reduced airway hyperresponsiveness in animal models [158]. Much more recently, Williams and colleagues [159] demonstrated the beneficial role of IL-25 monoclonal antibodies in increasing antiviral responses, which could translate into the prevention of viral-associated asthma exacerbations. Additionally, a combinational therapy involving IL-25 was suggested to induce beneficial effects.

In in vivo experiments performed by An et al. [160], researchers found that blockage of IL-25 with anti-TSLP reduced the presence of fibronectin, thus suggesting beneficial effects for fibrosis. Intranasal introduction of IL-25 in an experiment conducted on BALB/c mice showed increased expression of IL-5 and IL-13, resulting in local type-2 immunity skew, and consequently, pulmonary fibrosis [161]. In another study conducted on the same animal model, it was additionally proven that intranasal introduction of IL-25 leads to an increased release of Th2-type cytokines, as well as chemokines and collagen. In addition, there was an intensified production of epithelial mucus and hyperplasia within the smooth muscle cells of the respiratory tract [162]. In a study conducted on a mouse model of neutrophil-dominant allergic airway inflammation, it was shown that the administration of exogenous IL-25 had a positive effect on the rodents’ bodies. Oral therapy contributed to the reduction of macrophage polarization to the M1 type, as well as to the weakening of the expression of IL-1β, IL-17, IFN-γ, and TNFα. In addition, there was a decrease in the expression of IL-12 and IL-23 in the lungs [163]

Regarding the potential use of IL-25 as a biomarker, monitoring the level of this cytokine could perhaps distinguish potential responders to therapy. Specifically, significant reductions of serum IL-25 concentrations were noted in patients after a single dose of mepolizumab, a monoclonal antibody targeting IL-5 [164]. Nevertheless, this result was based on a small sample. In another study by Palacionyte and collaborators [165], researchers did not observe significant differences in IL-25 levels in T2-high asthma patients after 25 weeks of mepolizumab therapy. Different results were observed in the case of leukotriene inhibitors. Montelukast, a leukotriene receptor inhibitor used in the treatment of asthma, was associated with enhanced expression of IL-25 in airway epithelial cells. Importantly, this increase was prevented when montelukast was combined with corticosteroid, which demonstrates the benefits of combinational therapy [166]. The pathophysiology of asthma is also related to the presence of the M2 macrophages. These cells can be polarized into M1 and M2 subtypes, with evidence supporting the importance of the latter phenotype in this pulmonary disease [167]. IL-25 was also found to promote M2 macrophage polarization [168].

### 3.6. Cathelicidin and Defensins

Cathelicidin (LL-37) and defensins are other alarmins with immunoregulatory properties. However, their involvement in asthma pathophysiology seems to be less known than the previously described molecules due to the limited number of studies present within the literature. Furthermore, their role is context-dependent, as conflicting results have been previously demonstrated. To begin with, LL-37 regulates the expression of MMPs induced by TNFα in human bronchial epithelial cells. Specifically, in vitro experiments demonstrated that LL-37 reduces TNF-induced MMP-9 and MMP-13 levels. Therefore, it could limit airway remodeling induced by pro-inflammatory molecules in asthma [169]. Defensins are endogenous anti-microbial peptides that are divided into α and β subfamilies. The latter group is associated with epithelial cells. Pinkerton et al. [170] evaluated the protective effects of human β defensin 2 (hBD2) in the context of asthma. In mice models of allergic airway disease, intranasal administration of hBD2 reduced infiltration of immune cells within the airways. Importantly, the reduction also involved eosinophils and neutrophils. Accordingly, stimulation with hBD2 normalized the levels of several cytokines altered by house dust mite.

In a different study conducted by Borchers and colleagues [171], prophylactic oral use of hBD2 in BALB/c mice was investigated. The authors observed that this treatment did not change the immune cell count in bronchoalveolar lavage fluid but did reduce the levels of asthma-associated cytokines. In a clinical analysis, the expression of hBD2 was reduced in asthmatic children as compared to non-asthmatic controls [172]. This observation is in line with previous studies, as downregulation of hBD2 could represent one of the mechanisms involved in the pathophysiology of asthma. Furthermore, hBD1 and hBD3 demonstrated increased expression in asthma patients. In in vitro experiments, hBD3 silencing demonstrated positive effects in airway smooth muscle cells [172], thus suggesting hBD3 as a potential therapeutic target. Table 1 summarizes key mechanisms mediated by alarmins that are involved in the pathophysiology of asthma.

## 4. Conclusions and Future Perspectives

To conclude, alarmins are a large family of endogenous molecules that significantly mediate the activity of the immune system. Current evidence suggests their crucial involvement in the pathogenesis of asthma, as they enhance typical features related to the disease, including airway hyperreactivity, mucus overproduction, and mucus plug formation. However, the precise role of many alarmins is yet to be determined. While the evidence strongly suggests the pro-inflammatory role of HMGB1, IL-33, TSLP, and IL-25, different results were obtained regarding S100 proteins. While S100A9 and S100A4 promote the progression of asthma, S100A11 was suggested to relieve the pathological processes of the condition. Uncovering the role of alarmins in pathological processes opens a novel possibility of treatment, and accumulating studies are demonstrating the potential benefits of such treatment methods, such as the study involving tezepelumab. Moreover, identification of novel therapeutic targets that have complex immunoregulatory properties could help in suppressing asthma exacerbations, one of the hallmarks of the disease.

## Figures and Tables

**Table 1:** A summary of processes mediated by alarmins that play a role in the pathogenesis of asthma.

Alarmin	Involvement in the Pathophysiology of Asthma	Therapeutic Implications	References
HMGB1	↑ Eosinophils, IL-5, IL-13 in sputum; ↑ Th17, IL-4, IgG, IgE; ↑ TGF-β1, TNFα, TSLP, MM-9, VEGF, IL-6, IL-8, IL-17; Th17 and neutrophils—interaction with RAGE signaling; ↑ IL-1 β, IL-33—interaction with TLR4 signaling; ↑ TNFα, IL-1, IL-17, IFNγ—interaction with JAK/STAT signaling.	Inhaled glucocorticoid therapy—↓ HMGB1 in the squatum; HMGB1 neutralizing antibody—↓ HMGB and Th1, Th2, Th17’ vitamin D—↓ HMGB1 and suppressed inflammation within the respiratory tract in animal models; Astragaloside IV—↓ HMGB1 and RAGE signaling.	[38,41,42,46,47,48,51,58,59,60,61,65]
S100 proteins	↑ Neutrophils in the sputum; interaction with RAGE, MAPK signaling; ↑ Th2.	Dapagliflozin—↓ S100, leading to mitigated bronchospasm; Paquinimod—↓ inflammatory cells.	[67,79,80,93,94]
IL-33	↑ Eosinophils	Itepekimab—↑ lung function; Astegolimab—↓ exacerbation rate in patients suffering from low blood eosinophil asthma.	[102,110,111]
IL-25	↑ Fibrosis	Mepolizumab—↓ IL-25; Montelukast combined with corticosteroid—↓ IL-25	[155,158,159,160,165,166]
TSLP	↑ DC; ↑ Th2, IL-4, IL-5, IL-9, IL-13; ↑ Th17, neutrophils.	Tezepelumab—↓ IL-5, IL-13, IL-33, T2, eosinophils. Ecleralimab—↓ airway inflammation and bronchospasm; Lunsekimig—↓ TSLP, IL-13 Lipoxstatin-1 (Lip-1) —↓ TSLP.	[139,140,141,147,148,149]
LL-37	LL-37 reduces the expression of MMPs induced by TNF in bronchial epithelial cells.	-	[169]
Defensins	The expression of hBD2 is reduced in asthmatic children, while that of hBD1 and hBD3 increased.	The use of hBD2 as a treatment agent was shown to induce beneficial effects in animal studies. Silencing hBD3 could represent a promising treatment strategy in asthma.	[170,171,172]

↑—Increased levels or expression; ↓—Decreased levels or expression.

## Data Availability

Not applicable.

## Abbreviations

HMGB1	High-mobility group box-1
HMGN1	High-mobility group nucleosome-binding domain 1
HSP	Heat shock protein
TLR	Toll-like receptor
NF-κB	Nuclear factor kappa B
ILC2	Group 2 innate lymphoid cells
IL	Interleukin
EEP	Eosinophil extracellular traps
RAGE	Receptor for advanced glycation end products

## References

[1] Porsbjerg C., Melen E., Lehtimaki L., Shaw D. (2023). Asthma. Lancet.

[2] Cazzola M., Rogliani P., Ora J., Calzetta L., Matera M.G. (2022). Asthma and comorbidities: Recent advances. Pol. Arch. Intern. Med..

[3] Kuruvilla M.E., Lee F.E., Lee G.B. (2019). Understanding Asthma Phenotypes, Endotypes, and Mechanisms of Disease. Clin. Rev. Allergy Immunol..

[4] Taunk S.T., Cardet J.C., Ledford D.K. (2022). Clinical implications of asthma endotypes and phenotypes. Allergy Asthma Proc..

[5] Popović-Grle S., Štajduhar A., Lampalo M., Rnjak D. (2021). Biomarkers in Different Asthma Phenotypes. Genes.

[6] Ricciardolo F.L.M., Sprio A.E., Baroso A., Gallo F., Riccardi E., Bertolini F., Carriero V., Arrigo E., Ciprandi G. (2021). Characterization of T2-Low and T2-High Asthma Phenotypes in Real-Life. Biomedicines.

[7] Nguyen H., Nasir M. (2023). Management of Chronic Asthma in Adults. Prim. Care.

[8] Agache I., Akdis C.A., Akdis M., Canonica G.W., Casale T., Chivato T., Corren J., Chu D.K., Del Giacco S., Eiwegger T. (2021). EAACI Biologicals Guidelines-Recommendations for severe asthma. Allergy.

[9] Yang D., Han Z., Oppenheim J.J. (2017). Alarmins and immunity. Immunol. Rev..

[10] Bianchi M.E. (2007). DAMPs, PAMPs and alarmins: All we need to know about danger. J. Leukoc. Biol..

[11] Kielbowski K., Skorka P., Plewa P., Bakinowska E., Pawlik A. (2024). The Role of Alarmins in the Pathogenesis of Atherosclerosis and Myocardial Infarction. Curr. Issues Mol. Biol..

[12] Kielbowski K., Stanska W., Bakinowska E., Rusinski M., Pawlik A. (2024). The Role of Alarmins in the Pathogenesis of Rheumatoid Arthritis, Osteoarthritis, and Psoriasis. Curr. Issues Mol. Biol..

[13] Rao Z., Liu S., Li Z., Wang Q., Gao F., Peng H., Ren D., Zang Y., Li H., Li Y. (2024). Alarmin-loaded extracellular lipid droplets induce airway neutrophil infiltration during type 2 inflammation. Immunity.

[14] Werder R.B., Ullah M.A., Rahman M.M., Simpson J., Lynch J.P., Collinson N., Rittchen S., Rashid R.B., Sikder M.A.A., Handoko H.Y. (2022). Targeting the P2Y(13) Receptor Suppresses IL-33 and HMGB1 Release and Ameliorates Experimental Asthma. Am. J. Respir. Crit. Care Med..

[15] Fahy J.V. (2015). Type 2 inflammation in asthma--present in most, absent in many. Nat. Rev. Immunol..

[16] Wechsler M.E., Scelo G., Larenas-Linnemann D.E.S., Torres-Duque C.A., Maspero J., Tran T.N., Murray R.B., Martin N., Menzies-Gow A.N., Hew M. (2024). Association Between T2-related Comorbidities and Effectiveness of Biologics in Severe Asthma. Am. J. Respir. Crit. Care Med..

[17] Lu Y., Huang Y., Li J., Huang J., Zhang L., Feng J., Li J., Xia Q., Zhao Q., Huang L. (2021). Eosinophil extracellular traps drive asthma progression through neuro-immune signals. Nat. Cell Biol..

[18] Du X., Li F., Zhang C., Li N., Huang H., Shao Z., Zhang M., Zhan X., He Y., Ju Z. (2021). Eosinophil-derived chemokine (hCCL15/23, mCCL6) interacts with CCR1 to promote eosinophilic airway inflammation. Signal Transduct. Target. Ther..

[19] Choi Y., Kim Y.M., Lee H.R., Mun J., Sim S., Lee D.H., Pham D.L., Kim S.H., Shin Y.S., Lee S.W. (2020). Eosinophil extracellular traps activate type 2 innate lymphoid cells through stimulating airway epithelium in severe asthma. Allergy.

[20] Scott G., Asrat S., Allinne J., Keat Lim W., Nagashima K., Birchard D., Srivatsan S., Ajithdoss D.K., Oyejide A., Ben L.H. (2023). IL-4 and IL-13, not eosinophils, drive type 2 airway inflammation, remodeling and lung function decline. Cytokine.

[21] Olsthoorn S.E.M., van Krimpen A., Hendriks R.W., Stadhouders R. (2025). Chronic Inflammation in Asthma: Looking Beyond the Th2 Cell. Immunol. Rev..

[22] Cheema N.A., Castagna A., Ambrosani F., Argentino G., Friso S., Zurlo M., Beri R., Maule M., Vaia R., Senna G. (2025). Extracellular Vesicles in Asthma: Intercellular Cross-Talk in TH2 Inflammation. Cells.

[23] Kumagai T., Iwata A., Furuya H., Kato K., Okabe A., Toda Y., Kanai M., Fujimura L., Sakamoto A., Kageyama T. (2024). A distal enhancer of GATA3 regulates Th2 differentiation and allergic inflammation. Proc. Natl. Acad. Sci. USA.

[24] Baba R., Kabata H., Shirasaki Y., Kamatani T., Yamagishi M., Irie M., Watanabe R., Matsusaka M., Masaki K., Miyata J. (2022). Upregulation of IL-4 receptor signaling pathway in circulating ILC2s from asthma patients. J. Allergy Clin. Immunol. Glob..

[25] Matera M.G., Ora J., Calzetta L., Rogliani P., Cazzola M. (2023). Investigational anti IL-13 asthma treatments: A 2023 update. Expert. Opin. Investig. Drugs.

[26] Duffus E.K., Holguin F., Rastogi D. (2025). Non-T2 asthma. Curr. Opin. Pulm. Med..

[27] Goodwin G.H., Johns E.W. (1973). Isolation and characterisation of two calf-thymus chromatin non-histone proteins with high contents of acidic and basic amino acids. Eur. J. Biochem..

[28] Chen R., Kang R., Tang D. (2022). The mechanism of HMGB1 secretion and release. Exp. Mol. Med..

[29] Zhao Y., Li R. (2023). HMGB1 is a promising therapeutic target for asthma. Cytokine.

[30] Furci F., Murdaca G., Pelaia C., Imbalzano E., Pelaia G., Caminati M., Allegra A., Senna G., Gangemi S. (2023). TSLP and HMGB1: Inflammatory Targets and Potential Biomarkers for Precision Medicine in Asthma and COPD. Biomedicines.

[31] Yuan S., Liu Z., Xu Z., Liu J., Zhang J. (2020). High mobility group box 1 (HMGB1): A pivotal regulator of hematopoietic malignancies. J. Hematol. Oncol..

[32] Wang M., Gauthier A., Daley L., Dial K., Wu J., Woo J., Lin M., Ashby C., Mantell L.L. (2019). The Role of HMGB1, a Nuclear Damage-Associated Molecular Pattern Molecule, in the Pathogenesis of Lung Diseases. Antioxid. Redox Signal.

[33] Paudel Y.N., Angelopoulou E., Piperi C., Balasubramaniam V.R.M.T., Othman I., Shaikh M.F. (2019). Enlightening the role of high mobility group box 1 (HMGB1) in inflammation: Updates on receptor signalling. Eur. J. Pharmacol..

[34] Andersson U., Erlandsson-Harris H., Yang H., Tracey K.J. (2002). HMGB1 as a DNA-binding cytokine. J. Leukoc. Biol..

[35] Han Z., Junxu, Zhong N. (2003). Expression of matrix metalloproteinases MMP-9 within the airways in asthma. Respir. Med..

[36] Tang D., Kang R., Livesey K.M., Cheh C.W., Farkas A., Loughran P., Hoppe G., Bianchi M.E., Tracey K.J., Zeh H.J. (2010). Endogenous HMGB1 regulates autophagy. J. Cell Biol..

[37] Keyel P.A. (2014). How is inflammation initiated? Individual influences of IL-1, IL-18 and HMGB1. Cytokine.

[38] Yang Q., Li M., Hou Y., He H., Sun S. (2023). High-mobility group box 1 emerges as a therapeutic target for asthma. Immun. Inflamm. Dis..

[39] Chen X., Xu Y., Xiong P., Tan Z., Gong F., Hou X., Zheng F. (2018). Effects of mimicked acetylated HMGB1 on macrophages and dendritic cells. Mol. Med. Rep..

[40] Ibrahim Z.A., Armour C.L., Phipps S., Sukkar M.B. (2013). RAGE and TLRs: Relatives, friends or neighbours?. Mol. Immunol..

[41] Perkins T.N., Donnell M.L., Oury T.D. (2021). The axis of the receptor for advanced glycation endproducts in asthma and allergic airway disease. Allergy.

[42] Zhang H., Zhang J., Pan H., Yang K., Hu C. (2024). Astragaloside IV promotes the pyroptosis of airway smooth muscle cells in childhood asthma by suppressing HMGB1/RAGE axis to inactivate NF-κb pathway. Autoimmunity.

[43] Watanabe T., Asai K., Fujimoto H., Tanaka H., Kanazawa H., Hirata K. (2011). Increased levels of HMGB-1 and endogenous secretory RAGE in induced sputum from asthmatic patients. Respir. Med..

[44] Yang D., Chen Q., Yang H., Tracey K.J., Bustin M., Oppenheim J.J. (2007). High mobility group box-1 protein induces the migration and activation of human dendritic cells and acts as an alarmin. J. Leukoc. Biol..

[45] Xu S., Liu W., Zhang L., He Q., Ma C., Jiang J., Ye S., Ge L., Chen Z., Zhou L. (2023). High mobility group box 1 levels as potential predictors of asthma severity. Chin. Med. J..

[46] Manti S., Leonardi S., Parisi G.F., De Vivo D., Salpietro A., Spinuzza A., Arrigo T., Salpietro C., Cuppari C. (2017). High mobility group box 1: Biomarker of inhaled corticosteroid treatment response in children with moderate-severe asthma. Allergy Asthma Proc..

[47] Liu W., Li L., Piao Y., Wang Z., Dai L., Li Y., Piao H., Song Y., Cui Q., Wang C. (2025). Mechanism of action of miR-15b-5p in alleviating asthma airway remodeling through the HMGB1/TLR4/IL-33 signaling axis. Int. Immunopharmacol..

[48] Shim E.J., Chun E., Lee H.S., Bang B.R., Kim T.W., Cho S.H., Min K.U., Park H.W. (2012). The role of high-mobility group box-1 (HMGB1) in the pathogenesis of asthma. Clin. Exp. Allergy.

[49] Stenfeldt A.L., Wennerås C. (2004). Danger signals derived from stressed and necrotic epithelial cells activate human eosinophils. Immunology.

[50] Brandt E.B., Lewkowich I.P. (2019). RAGE-induced asthma: A role for the receptor for advanced glycation end-products in promoting allergic airway disease. J. Allergy Clin. Immunol..

[51] Sun J., Jiang Y., Li L., Li R., Ling F., Du X., Han Q., Chu S., Liang Y., Mai L. (2024). HMGB1/RAGE Signaling Regulates Th17/IL-17 and Its Role in Bronchial Epithelial-Mesenchymal Transformation. Curr. Mol. Med..

[52] Liang Y., Hou C., Kong J., Wen H., Zheng X., Wu L., Huang H., Chen Y. (2015). HMGB1 binding to receptor for advanced glycation end products enhances inflammatory responses of human bronchial epithelial cells by activating p38 MAPK and ERK1/2. Mol. Cell Biochem..

[53] Popa C., Netea M.G., van Riel P.L., van der Meer J.W., Stalenhoef A.F. (2007). The role of TNF-alpha in chronic inflammatory conditions, intermediary metabolism, and cardiovascular risk. J. Lipid Res..

[54] Asai K., Kanazawa H., Kamoi H., Shiraishi S., Hirata K., Yoshikawa J. (2003). Increased levels of vascular endothelial growth factor in induced sputum in asthmatic patients. Clin. Exp. Allergy.

[55] Ying S., O’Connor B., Ratoff J., Meng Q., Fang C., Cousins D., Zhang G., Gu S., Gao Z., Shamji B. (2008). Expression and cellular provenance of thymic stromal lymphopoietin and chemokines in patients with severe asthma and chronic obstructive pulmonary disease. J. Immunol..

[56] Zhou Y., Jiang Y.Q., Wang W.X., Zhou Z.X., Wang Y.G., Yang L., Ji Y.L. (2012). HMGB1 and RAGE levels in induced sputum correlate with asthma severity and neutrophil percentage. Hum. Immunol..

[57] Davies D.E. (2001). The bronchial epithelium in chronic and severe asthma. Curr. Allergy Asthma Rep..

[58] Yamasaki A., Okazaki R., Harada T. (2022). Neutrophils and Asthma. Diagnostics.

[59] Chang J., Xia Y., Wasserloos K., Deng M., Blose K.J., Vorp D.A., Turnquist H.R., Billiar T.R., Pitt B.A., Zhang M.Z. (2017). Cyclic stretch induced IL-33 production through HMGB1/TLR-4 signaling pathway in murine respiratory epithelial cells. PLoS ONE.

[60] Fu J., Lin S.H., Wang C.J., Li S.Y., Feng X.Y., Liu Q., Xu F. (2016). HMGB1 regulates IL-33 expression in acute respiratory distress syndrome. Int. Immunopharmacol..

[61] Lee C.C., Lai Y.T., Chang H.T., Liao J.W., Shyu W.C., Li C.Y., Wang C.N. (2013). Inhibition of high-mobility group box 1 in lung reduced airway inflammation and remodeling in a mouse model of chronic asthma. Biochem. Pharmacol..

[62] Hwang Y.H., Lee Y., Paik M.J., Yee S.T. (2019). Inhibitions of HMGB1 and TLR4 alleviate DINP-induced asthma in mice. Toxicol. Res..

[63] Hou C., Kong J., Liang Y., Huang H., Wen H., Zheng X., Wu L., Chen Y. (2015). HMGB1 contributes to allergen-induced airway remodeling in a murine model of chronic asthma by modulating airway inflammation and activating lung fibroblasts. Cell Mol. Immunol..

[64] Tang H., Zhao H., Song J., Dong H., Yao L., Liang Z., LV Y., Zou F., Cai S. (2014). Ethyl pyruvate decreases airway neutrophil infiltration partly through a high mobility group box 1-dependent mechanism in a chemical-induced murine asthma model. Int. Immunopharmacol..

[65] Zhang H., Yang N., Wang T., Dai B., Shang Y. (2018). Vitamin D reduces inflammatory response in asthmatic mice through HMGB1/TLR4/NF-κB signaling pathway. Mol. Med. Rep..

[66] Moore B.W. (1965). A soluble protein characteristic of the nervous system. Biochem. Biophys. Res. Commun..

[67] Sattar Z., Lora A., Jundi B., Railwah C., Geraghty P. (2021). The S100 Protein Family as Players and Therapeutic Targets in Pulmonary Diseases. Pulm. Med..

[68] Kozlyuk N., Monteith A.J., Garcia V., Damo S.M., Skaar E.P., Chazin W.J. (2019). S100 Proteins in the Innate Immune Response to Pathogens. Methods Mol. Biol..

[69] Gonzalez L.L., Garrie K., Turner M.D. (2020). Role of S100 proteins in health and disease. Biochim. Biophys. Acta Mol. Cell Res..

[70] Marenholz I., Heizmann C.W., Fritz G. (2004). S100 proteins in mouse and man: From evolution to function and pathology (including an update of the nomenclature). Biochem. Biophys. Res. Commun..

[71] Singh P., Ali S.A. (2022). Multifunctional Role of S100 Protein Family in the Immune System: An Update. Cells.

[72] Cerón J.J., Ortín-Bustillo A., López-Martínez M.J., Martínez-Subiela S., Eckersall P.D., Tecles F., Tvarijonaviciute A., Muñoz-Prieto A. (2023). S-100 Proteins: Basics and Applications as Biomarkers in Animals with Special Focus on Calgranulins (S100A8, A9, and A12). Biology.

[73] Gilston B.A., Skaar E.P., Chazin W.J. (2016). Binding of transition metals to S100 proteins. Sci. China Life Sci..

[74] Wheeler L.C., Donor M.T., Prell J.S., Harms M.J. (2016). Multiple Evolutionary Origins of Ubiquitous Cu^2+^ and Zn^2+^ Binding in the S100 Protein Family. PLoS ONE.

[75] Sedaghat F., Notopoulos A. (2008). S100 protein family and its application in clinical practice. Hippokratia.

[76] Zimmer D.B., Eubanks J.O., Ramakrishnan D., Criscitiello M.F. (2013). Evolution of the S100 family of calcium sensor proteins. Cell Calcium.

[77] Schenten V., Plançon S., Jung N., Hann J., Bueb J.L., Bréchard S., Tschirhart E.J., Tolle F. (2018). Secretion of the Phosphorylated Form of S100A9 from Neutrophils Is Essential for the Proinflammatory Functions of Extracellular S100A8/A9. Front. Immunol..

[78] Sprenkeler E.G.G., Zandstra J., van Kleef N.D., Goetschalckx I., Verstegen B., Aarts C.E.M., Janssen H., Tool A.T.J., van Mierlo G., van Bruggen R. (2022). S100A8/A9 Is a Marker for the Release of Neutrophil Extracellular Traps and Induces Neutrophil Activation. Cells.

[79] Quoc Q.L., Choi Y., Thi Bich T.C., Yang E.M., Shin Y.S., Park H.S. (2021). S100A9 in adult asthmatic patients: A biomarker for neutrophilic asthma. Exp. Mol. Med..

[80] Huang C., Zheng D., Fu C., Cai Z., Zhang H., Xie Z., Luo L., Li H., Huang Y., Chen J. (2023). Secreted S100A4 causes asthmatic airway epithelial barrier dysfunction induced by house dust mite extracts via activating VEGFA/VEGFR2 pathway. Environ. Toxicol..

[81] Shihui M., Shirong Y., Jing L., Jingjing H., Tongqian W., Tian T., Chenyu W., Fang Y. (2024). S100A4 reprofiles lipid metabolism in mast cells via RAGE and PPAR-γ signaling pathway. Int. Immunopharmacol..

[82] Wu Y., Zhang W., Gunst S.J. (2020). S100A4 is secreted by airway smooth muscle tissues and activates inflammatory signaling pathways via receptors for advanced glycation end products. Am. J. Physiol. Lung Cell Mol. Physiol..

[83] Liu M., Liu S., Li F., Li C., Chen S., Gao X., Wang X. (2023). The miR-124-3p regulates the allergic airway inflammation and remodeling in an ovalbumin-asthmatic mouse model by inhibiting S100A4. Immun. Inflamm. Dis..

[84] Wu T., Ma L., Jin X., He J., Chen K., Zhang D., Yuan R., Yang J., Zhong Q., Zhou H. (2021). S100A4 Is Critical for a Mouse Model of Allergic Asthma by Impacting Mast Cell Activation. Front. Immunol..

[85] Cheng M., Shi Y.L., Shang P.P., Chen Y.J., Xu Y.D. (2022). Inhibitory Effect of S100A11 on Airway Smooth Muscle Contraction and Airway Hyperresponsiveness. Curr. Med. Sci..

[86] Gray R.D., MacGregor G., Noble D., Imrie M., Dewar M., Boyd A.C., Innes J.A., Porteous D.J., Greening A.P. (2008). Sputum proteomics in inflammatory and suppurative respiratory diseases. Am. J. Respir. Crit. Care Med..

[87] Lee T.H., Chang H.S., Bae D.J., Song H.J., Kim M.S., Park J.S., Jun J.A., Lee S.Y., Uh S.T., Kim S.H. (2017). Role of S100A9 in the development of neutrophilic inflammation in asthmatics and in a murine model. Clin. Immunol..

[88] Eguíluz-Gracia I., Malmstrom K., Dheyauldeen S.A., Lohi J., Sajantila A., Aaløkken R., Sundaram A.Y.M., Gilfillan G.D., Makela M., Baekkevold E.S. (2018). Monocytes accumulate in the airways of children with fatal asthma. Clin. Exp. Allergy.

[89] Aoki T., Matsumoto Y., Hirata K., Ochiai K., Okada M., Ichikawa K., Shibasaki M., Arinami T., Sumazaki R., Noguchi E. (2009). Expression profiling of genes related to asthma exacerbations. Clin. Exp. Allergy.

[90] Wang C.H., Punde T.H., Huang C.D., Chou P.C., Huang T.T., Wu W.H., Liu C.H., Chung K.F., Kuo H.P. (2015). Fibrocyte trafficking in patients with chronic obstructive asthma and during an acute asthma exacerbation. J. Allergy Clin. Immunol..

[91] Yang Z., Yan W.X., Cai H., Tedla N., Armishaw C., Di Girolamo N., Wang H.W., Hampartzoumian T., Simpson J.L., Gibson P.G. (2007). S100A12 provokes mast cell activation: A potential amplification pathway in asthma and innate immunity. J. Allergy Clin. Immunol..

[92] Tsubokawa D., Satoh M. (2022). Strongyloides venezuelensis-derived venestatin ameliorates asthma pathogenesis by suppressing receptor for advanced glycation end-products-mediated signaling. Pulm. Pharmacol. Ther..

[93] Tabaa M.M.E., Fattah A.M.K., Shaalan M., Rashad E., El Mahdy N.A. (2022). Dapagliflozin mitigates ovalbumin-prompted airway inflammatory-oxidative successions and associated bronchospasm in a rat model of allergic asthma. Expert. Opin. Ther. Targets.

[94] Lee J.U., Park J.S., Jun J.A., Kim M.K., Chang H.S., Baek D.G., Song H.J., Kim M.S., Park C.S. (2021). Inhibitory Effect of Paquinimod on a Murine Model of Neutrophilic Asthma Induced by Ovalbumin with Complete Freund’s Adjuvant. Can. Respir. J..

[95] Huang X., Qu D., Liang Y., Huang Q., Li M., Hou C. (2019). Elevated S100A4 in asthmatics and an allergen-induced mouse asthma model. J. Cell Biochem..

[96] Pinto S.M., Subbannayya Y., Rex D.A.B., Raju R., Chatterjee O., Advani J., Radhakrishnan A., Keshava Prasad T.S., Wani M.R., Pandey A. (2018). A network map of IL-33 signaling pathway. J. Cell Commun. Signal..

[97] Schmitz J., Owyang A., Oldham E., Song Y., Murphy E., McClanahan T.K., Zurawski G., Moshrefi M., Qin J., Li X. (2005). IL-33, an interleukin-1-like cytokine that signals via the IL-1 receptor-related protein ST2 and induces T helper type 2-associated cytokines. Immunity.

[98] Liang Y., Wang X., Wang H., Yang W., Yi P., Soong L., Cong Y., Cai J., Fan X., Sun J. (2022). IL-33 activates mTORC1 and modulates glycolytic metabolism in CD8(+) T cells. Immunology.

[99] Marx A.F., Kallert S.M., Brunner T.M., Villegas J.A., Geier F., Fixemer J., Abreu-Mota T., Reuther P., Bonilla W.V., Fadejeva J. (2023). The alarmin interleukin-33 promotes the expansion and preserves the stemness of Tcf-1(+) CD8(+) T cells in chronic viral infection. Immunity.

[100] Zou L., Dang W., Tao Y., Zhao H., Yang B., Xu X., Li Y. (2023). The Il-33/St2 Axis Promotes Acute Respiratory Distress Syndrome by Natural Killer T Cells. Shock..

[101] Mok M.Y., Luo C.Y., Huang F.P., Kong W.Y., Chan G.C.F. (2023). IL-33 Orchestrated the Interaction and Immunoregulatory Functions of Alternatively Activated Macrophages and Regulatory T Cells In Vitro. J. Immunol..

[102] Ketelaar M.E., Portelli M.A., Dijk F.N., Shrine N., Faiz A., Vermeulen C.J., Xu C.J., Hankinson J., Bhaker S., Henry A.P. (2021). Phenotypic and functional translation of IL33 genetics in asthma. J. Allergy Clin. Immunol..

[103] Ji Y., Wang E., Mohammed M.T., Hameed N., Christodoulou M.I., Liu X., Zhou W., Fang Z., Jia N., Yu H. (2024). Selective production of IL-33-neutralizing autoantibody ameliorates asthma responses and severity. Clin. Immunol..

[104] Poulsen N.N., Bjerregaard A., Khoo S.K., Laing I.A., Le Souëf P., Backer V., Rapley L., Cohen S.E., Barrett L., Thompson P. (2018). Airway Interleukin-33 and type 2 cytokines in adult patients with acute asthma. Respir. Med..

[105] Du J., Liu Y., Lan G., Zhou Y., Ni Y., Liao K., Zheng F., Cheng Q., Shi G., Su X. (2023). PTRF-IL33-ZBP1 signaling mediating macrophage necroptosis contributes to HDM-induced airway inflammation. Cell Death Dis..

[106] Schuijs M.J., Brenis Gomez C.M., Bick F., Van Moorleghem J., Vanheerswynghels M., van Loo G., Beyaert R., Voehringer D., Locksley R.M., Hammad H. (2024). Interleukin-33-activated basophils promote asthma by regulating Th2 cell entry into lung tissue. J. Exp. Med..

[107] Wu M., Zheng X., Huang J., Hu X. (2021). Association of. Front. Cell Dev. Biol..

[108] Badi Y.E., Salcman B., Taylor A., Rana B., Kermani N.Z., Riley J.H., Worsley S., Mumby S., Dahlen S.E., Cousins D. (2023). IL1RAP expression and the enrichment of IL-33 activation signatures in severe neutrophilic asthma. Allergy.

[109] van der Ploeg E.K., Krabbendam L., Vroman H., van Nimwegen M., de Bruijn M.J.W., de Boer G.M., Bergen I.M., Kool M., Tramper-Standers G.A., Braunstahl G.J. (2023). Type-2 CD8(+) T-cell formation relies on interleukin-33 and is linked to asthma exacerbations. Nat. Commun..

[110] Wechsler M.E., Ruddy M.K., Pavord I.D., Israel E., Rabe K.F., Ford L.B., Maspero J.F., Abdulai R.M., Hu C.C., Martincova R. (2021). Efficacy and Safety of Itepekimab in Patients with Moderate-to-Severe Asthma. N. Engl. J. Med..

[111] Kelsen S.G., Agache I.O., Soong W., Israel E., Chupp G.L., Cheung D.S., Theess W., Yang X., Staton T.L., Choy D.F. (2021). Astegolimab (anti-ST2) efficacy and safety in adults with severe asthma: A randomized clinical trial. J. Allergy Clin. Immunol..

[112] Liu H., Jing X., Yu L., Jiang Z., Lu Y., Peng J., Xu X., Liu H., Li R., Tang H. (2025). Metformin alleviates inflammatory responses in acute allergic asthma by inhibiting ILC2s function. Int. Immunopharmacol..

[113] Friend S.L., Hosier S., Nelson A., Foxworthe D., Williams D.E., Farr A. (1994). A thymic stromal cell line supports in vitro development of surface IgM+ B cells and produces a novel growth factor affecting B and T lineage cells. Exp. Hematol..

[114] Zhong J., Sharma J., Raju R., Palapetta S.M., Prasad T.S., Huang T.C., Yoda A., Tyner J.W., van Bodegom D., Weinstock D.M. (2014). TSLP signaling pathway map: A platform for analysis of TSLP-mediated signaling. Database.

[115] Sebastian K., Borowski A., Kuepper M., Friedrich K. (2008). Signal transduction around thymic stromal lymphopoietin (TSLP) in atopic asthma. Cell Commun. Signal..

[116] Smolinska S., Antolín-Amérigo D., Popescu F.D., Jutel M. (2023). Thymic Stromal Lymphopoietin (TSLP), Its Isoforms and the Interplay with the Epithelium in Allergy and Asthma. Int. J. Mol. Sci..

[117] Verstraete K., Peelman F., Braun H., Lopez J., Van Rompaey D., Dansercoer A., Vandenberghe I., Pauwels K., Tavernier J., Lambrecht B.N. (2017). Structure and antagonism of the receptor complex mediated by human TSLP in allergy and asthma. Nat. Commun..

[118] Tsilingiri K., Fornasa G., Rescigno M. (2017). Thymic Stromal Lymphopoietin: To Cut a Long Story Short. Cell Mol. Gastroenterol. Hepatol..

[119] Park J.H., Jeong D.Y., Peyrin-Biroulet L., Eisenhut M., Shin J.I. (2017). Insight into the role of TSLP in inflammatory bowel diseases. Autoimmun. Rev..

[120] Xie Y., Takai T., Chen X., Okumura K., Ogawa H. (2012). Long TSLP transcript expression and release of TSLP induced by TLR ligands and cytokines in human keratinocytes. J. Dermatol. Sci..

[121] Borowski A., Vetter T., Kuepper M., Wohlmann A., Krause S., Lorenzen T., Virchow J.C., Luttmann W., Friedrich K. (2013). Expression analysis and specific blockade of the receptor for human thymic stromal lymphopoietin (TSLP) by novel antibodies to the human TSLPRα receptor chain. Cytokine.

[122] Matera M.G., Rogliani P., Calzetta L., Cazzola M. (2020). TSLP Inhibitors for Asthma: Current Status and Future Prospects. Drugs.

[123] Scheeren F.A., van Lent A.U., Nagasawa M., Weijer K., Spits H., Legrand N., Blom B. (2010). Thymic stromal lymphopoietin induces early human B-cell proliferation and differentiation. Eur. J. Immunol..

[124] Allakhverdi Z., Comeau M.R., Jessup H.K., Yoon B.R., Brewer A., Chartier S., Paquette N., Ziegler S.F., Sarfati M., Delespesse G. (2007). Thymic stromal lymphopoietin is released by human epithelial cells in response to microbes, trauma, or inflammation and potently activates mast cells. J. Exp. Med..

[125] Siracusa M.C., Saenz S.A., Hill D.A., Kim B.S., Headley M.B., Doering T.A., Wherry E.J., Jessup H.K., Siegel L.A., Kambayashi T. (2011). TSLP promotes interleukin-3-independent basophil haematopoiesis and type 2 inflammation. Nature.

[126] Hui C.C., Rusta-Sallehy S., Asher I., Heroux D., Denburg J.A. (2014). The effects of thymic stromal lymphopoietin and IL-3 on human eosinophil-basophil lineage commitment: Relevance to atopic sensitization. Immun. Inflamm. Dis..

[127] Leyva-Castillo J.M., Hener P., Michea P., Karasuyama H., Chan S., Soumelis V., Li M. (2013). Skin thymic stromal lymphopoietin initiates Th2 responses through an orchestrated immune cascade. Nat. Commun..

[128] Varricchi G., Pecoraro A., Marone G., Criscuolo G., Spadaro G., Genovese A. (2018). Thymic Stromal Lymphopoietin Isoforms, Inflammatory Disorders, and Cancer. Front. Immunol..

[129] Rochman Y., Kashyap M., Robinson G.W., Sakamoto K., Gomez-Rodriguez J., Wagner K.U., Leonard W.J. (2010). Thymic stromal lymphopoietin-mediated STAT5 phosphorylation via kinases JAK1 and JAK2 reveals a key difference from IL-7-induced signaling. Proc. Natl. Acad. Sci. USA.

[130] Yu X., Li H., Ren X. (2012). Signaling cascades initiated by TSLP-mediated signals in different cell types. Cell Immunol..

[131] Parnes J.R., Molfino N.A., Colice G., Martin U., Corren J., Menzies-Gow A. (2022). Targeting TSLP in Asthma. J. Asthma Allergy.

[132] Chorvinsky E., Nino G., Salka K., Gaviria S., Gutierrez M.J., Pillai D.K. (2022). TSLP bronchoalveolar lavage levels at baseline are linked to clinical disease severity and reduced lung function in children with asthma. Front. Pediatr..

[133] Canè L., Poto R., Palestra F., Pirozzi M., Parashuraman S., Iacobucci I., Ferrara A.L., La Rocca A., Mercadante E., Pucci P. (2024). TSLP is localized in and released from human lung macrophages activated by T2-high and T2-low stimuli: Relevance in asthma and COPD. Eur. J. Intern. Med..

[134] Murrison L.B., Ren X., Preusse K., He H., Kroner J., Chen X., Jenkins S., Johansson E., Biagini J.M., Weirauch M.T. (2022). TSLP disease-associated genetic variants combined with airway TSLP expression influence asthma risk. J. Allergy Clin. Immunol..

[135] Ying S., O’Connor B., Ratoff J., Meng Q., Mallett K., Cousins D., Robinson D., Zhang G., Zhao J., Lee T.H. (2005). Thymic stromal lymphopoietin expression is increased in asthmatic airways and correlates with expression of Th2-attracting chemokines and disease severity. J. Immunol..

[136] Wang W., Li Y., Lv Z., Chen Y., Huang K., Corrigan C.J., Ying S. (2018). Bronchial Allergen Challenge of Patients with Atopic Asthma Triggers an Alarmin (IL-33, TSLP, and IL-25) Response in the Airways Epithelium and Submucosa. J. Immunol..

[137] Paplińska-Goryca M., Nejman-Gryz P., Proboszcz M., Kwiecień I., Hermanowicz-Salamon J., Grabczak E.M., Krenke R. (2020). Expression of TSLP and IL-33 receptors on sputum macrophages of asthma patients and healthy subjects. J. Asthma.

[138] Toki S., Goleniewska K., Zhang J., Zhou W., Newcomb D.C., Zhou B., Kita H., Boyd K.L., Peebles R.S. (2020). TSLP and IL-33 reciprocally promote each other’s lung protein expression and ILC2 receptor expression to enhance innate type-2 airway inflammation. Allergy.

[139] Pelaia C., Pelaia G., Crimi C., Maglio A., Gallelli L., Terracciano R., Vatrella A. (2021). Tezepelumab: A Potential New Biological Therapy for Severe Refractory Asthma. Int. J. Mol. Sci..

[140] Sverrild A., Cerps S., Nieto-Fontarigo J.J., Ramu S., Hvidtfeldt M., Menzel M., Kearley J., Griffiths J.M., Parnes J.R., Porsbjerg C. (2024). Tezepelumab decreases airway epithelial IL-33 and T2-inflammation in response to viral stimulation in patients with asthma. Allergy.

[141] Corren J., Pham T.H., Garcia Gil E., Sałapa K., Ren P., Parnes J.R., Colice G., Griffiths J.M. (2022). Baseline type 2 biomarker levels and response to tezepelumab in severe asthma. Allergy.

[142] Diver S., Khalfaoui L., Emson C., Wenzel S.E., Menzies-Gow A., Wechsler M.E., Johnston J., Molfino N., Parnes J.R., Megally A. (2021). Effect of tezepelumab on airway inflammatory cells, remodelling, and hyperresponsiveness in patients with moderate-to-severe uncontrolled asthma (CASCADE): A double-blind, randomised, placebo-controlled, phase 2 trial. Lancet Respir. Med..

[143] Greig R., Chan R., Fardon T.C., Lipworth B.J. (2025). Real-world effects of tezepelumab on small airway dysfunction in severe refractory asthma. Ann. Allergy Asthma Immunol..

[144] Carpagnano G.E., Dragonieri S., Resta E., Lulaj E., Montagnolo F., Portacci A., Magaletti P., Soccio P., Lacedonia D., Scioscia G. (2025). Short-term Tezepelumab effectiveness in patients with severe asthma: A multicenter study. J. Asthma.

[145] Qin J., Wang G., Han D. (2025). Long-term safety of tezepelumab in patients with asthma: A systematic review and meta-analysis of randomized controlled trials. J. Asthma.

[146] Portacci A., Scioscia G., Dragonieri S., Aliani M., Lulaj E., Montagnolo F., Magaletti P., Soccio P., Salerno L., Lacedonia D. (2025). The impact of tezepelumab therapy on perceived asthma triggers: A multicenter real-life study. J. Asthma.

[147] Gauvreau G.M., Hohlfeld J.M., FitzGerald J.M., Boulet L.P., Cockcroft D.W., Davis B.E., Korn S., Kornmann O., Leigh R., Mayers I. (2023). Inhaled anti-TSLP antibody fragment, ecleralimab, blocks responses to allergen in mild asthma. Eur. Respir. J..

[148] Deiteren A., Bontinck L., Conickx G., Vigan M., Dervaux N., Gassiot M., Bas S., Suratt B., Staudinger H., Krupka E. (2024). A first-in-human, single and multiple dose study of lunsekimig, a novel anti-TSLP/anti-IL-13 NANOBODY^®^ compound, in healthy volunteers. Clin. Transl. Sci..

[149] Bao C., Liu C., Liu Q., Hua L., Hu J., Li Z., Xu S. (2022). Liproxstatin-1 alleviates LPS/IL-13-induced bronchial epithelial cell injury and neutrophilic asthma in mice by inhibiting ferroptosis. Int. Immunopharmacol..

[150] Cheng D.T., Ma C., Niewoehner J., Dahl M., Tsai A., Zhang J., Gonsiorek W., Apparsundaram S., Pashine A., Ravindran P. (2013). Thymic stromal lymphopoietin receptor blockade reduces allergic inflammation in a cynomolgus monkey model of asthma. J. Allergy Clin. Immunol..

[151] Murphy M.B., Vitale L., O’Neill T., Maurer D.M., Malenchek L., Crocker A., Patterson C., Mills-Chen L., Saley V., Antczak N.M. (2025). Dual Inhibition of Mast Cells and Thymic Stromal Lymphopoietin Using a Novel Bispecific Antibody, CDX-622. Allergy.

[152] Deiteren A., Krupka E., Bontinck L., Imberdis K., Conickx G., Bas S., Patel N., Staudinger H.W., Suratt B.T. (2025). A proof-of-mechanism trial in asthma with lunsekimig, a bispecific NANOBODY molecule. Eur. Respir. J..

[153] Zeng Z., Ruan Y., Ying H., Wang J., Wang H., Chen S. (2025). Baicalin Attenuates Type 2 Immune Responses in a Mouse Allergic Asthma Model through Inhibiting the Production of Thymic Stromal Lymphopoietin. Int. Arch. Allergy Immunol..

[154] Xu M., Dong C. (2017). IL-25 in allergic inflammation. Immunol. Rev..

[155] Yao X., Chen Q., Wang X., Liu X., Zhang L. (2023). IL-25 induces airway remodeling in asthma by orchestrating the phenotypic changes of epithelial cell and fibrocyte. Respir. Res..

[156] Peng B., Sun L., Zhang M., Yan H., Shi G., Xia Z., Dai R., Tang W. (2022). Role of IL-25 on Eosinophils in the Initiation of Th2 Responses in Allergic Asthma. Front. Immunol..

[157] Zhang K., Feng Y., Liang Y., Wu W., Chang C., Chen D., Chen S., Gao J., Chen G., Yi L. (2021). Epithelial miR-206 targets CD39/extracellular ATP to upregulate airway IL-25 and TSLP in type 2-high asthma. JCI Insight.

[158] Ballantyne S.J., Barlow J.L., Jolin H.E., Nath P., Williams A.S., Chung K.F., Sturton G., Wong S.H., McKenzie A.N. (2007). Blocking IL-25 prevents airway hyperresponsiveness in allergic asthma. J. Allergy Clin. Immunol..

[159] Williams T.C., Loo S.L., Nichol K.S., Reid A.T., Veerati P.C., Esneau C., Wark P.A.B., Grainge C.L., Knight D.A., Vincent T. (2022). IL-25 blockade augments antiviral immunity during respiratory virus infection. Commun. Biol..

[160] An G., Wang W., Zhang X., Huang Q., Li Q., Chen S., Du X., Corrigan C.J., Huang K., Wang W. (2020). Combined blockade of IL-25, IL-33 and TSLP mediates amplified inhibition of airway inflammation and remodelling in a murine model of asthma. Respirology.

[161] Xu X., Luo S., Li B., Dai H., Zhang J. (2019). IL-25 contributes to lung fibrosis by directly acting on alveolar epithelial cells and fibroblasts. Exp. Biol. Med..

[162] Yao X.J., Huang K.W., Li Y., Zhang Q., Wang J.J., Wang W., Liu J., Lv Z., An Y.Q., Ding Y.Z. (2014). Direct comparison of the dynamics of IL-25- and ‘allergen’-induced airways inflammation, remodelling and hypersensitivity in a murine asthma model. Clin. Exp. Allergy.

[163] Chang C., Chen G., Wu W., Chen D., Chen S., Gao J., Feng Y., Zhen G. (2023). Exogenous IL-25 ameliorates airway neutrophilia via suppressing macrophage M1 polarization and the expression of IL-12 and IL-23 in asthma. Respir. Res..

[164] Kalinauskaite-Zukauske V., Januskevicius A., Janulaityte I., Miliauskas S., Malakauskas K. (2019). Serum Levels of Epithelial-Derived Cytokines as Interleukin-25 and Thymic Stromal Lymphopoietin after a Single Dose of Mepolizumab in Patients with Severe Non-Allergic Eosinophilic Asthma: A Short Report. Can. Respir. J..

[165] Palacionyte J., Januskevicius A., Vasyle E., Rimkunas A., Miliauskas S., Malakauskas K. (2024). Clinical Remission Criteria and Serum Levels of Type 2 Inflammation Mediators during 24 Weeks of Treatment with the Anti-IL-5 Drug Mepolizumab in Patients with T2-High Severe Asthma. Diagnostics.

[166] Tsai M.L., Tsai M.K., Tsai Y.G., Lin Y.C., Hsu Y.L., Chen Y.T., Lin Y.C., Hung C.H. (2023). Montelukast Increased IL-25, IL-33, and TSLP via Epigenetic Regulation in Airway Epithelial Cells. Int. J. Mol. Sci..

[167] Arora S., Dev K., Agarwal B., Das P., Syed M.A. (2018). Macrophages: Their role, activation and polarization in pulmonary diseases. Immunobiology.

[168] Tsai M.L., Tsai Y.G., Lin Y.C., Hsu Y.L., Chen Y.T., Tsai M.K., Liao W.T., Lin Y.C., Hung C.H. (2021). IL-25 Induced ROS-Mediated M2 Macrophage Polarization via AMPK-Associated Mitophagy. Int. J. Mol. Sci..

[169] Altieri A., Marshall C.L., Ramotar P., Lloyd D., Hemshekhar M., Spicer V., van der Does A.M., Mookherjee N. (2024). Human Host Defense Peptide LL-37 Suppresses TNFalpha-Mediated Matrix Metalloproteinases MMP9 and MMP13 in Human Bronchial Epithelial Cells. J. Innate Immun..

[170] Pinkerton J.W., Kim R.Y., Koeninger L., Armbruster N.S., Hansbro N.G., Brown A.C., Jayaraman R., Shen S., Malek N., Cooper M.A. (2021). Human beta-defensin-2 suppresses key features of asthma in murine models of allergic airways disease. Clin. Exp. Allergy.

[171] Borchers N.S., Santos-Valente E., Toncheva A.A., Wehkamp J., Franke A., Gaertner V.D., Nordkild P., Genuneit J., Jensen B.A.H., Kabesch M. (2021). Human beta-Defensin 2 Mutations Are Associated with Asthma and Atopy in Children and Its Application Prevents Atopic Asthma in a Mouse Model. Front. Immunol..

[172] Chen G., Zheng Y., Wu N., Yang X., Qu S. (2024). Human beta defensin 3 knockdown inhibits the proliferation and migration of airway smooth muscle cells through regulating the PI3K/AKT signaling pathway. Mol. Immunol..

